# Cannabidiol for Scan-Related Anxiety in Women With Advanced Breast Cancer: A Randomized Clinical Trial

**DOI:** 10.1001/jamanetworkopen.2024.50391

**Published:** 2024-12-16

**Authors:** Manan M. Nayak, Peter Chai, Paul J. Catalano, William F. Pirl, James A. Tulsky, Stephanie C. Tung, Nancy U. Lin, Nicole Andrade, Sabrina Johns, Clint Vaz, Melissa Hughes, Ilana M. Braun

**Affiliations:** 1 Department of Supportive Oncology, Dana-Farber Cancer Institute, Boston, Massachusetts; 2 Phyllis F. Cantor Center for Research in Nursing and Patient Care Services, Dana-Farber Cancer Institute, Boston, Massachusetts; 3 Department of Medicine, Harvard Medical School, Boston, Massachusetts; 4 Department of Emergency Medicine, Brigham and Women’s Hospital, Boston, Massachusetts; 5 Department of Mechanical Engineering, Massachusetts Institute of Technology, Cambridge; 6 The Fenway Institute, Boston, Massachusetts; 7 Department of Biostatistics, Harvard T.H. Chan School of Public Health, Boston, Massachusetts; 8 Department of Data Science, Dana-Farber Cancer Institute, Boston, Massachusetts; 9 Department of Psychiatry, Harvard Medical School, Boston, Massachusetts; 10 Department of Psychiatry, Brigham and Women’s Hospital, Boston, Massachusetts; 11 Department of Medicine, Brigham and Women’s Hospital, Boston, Massachusetts; 12 Department of Medical Oncology, Dana-Farber Cancer Institute, Boston, Massachusetts; 13 Department of Internal Medicine, New York Medical College at St Clare’s and St Mary’s Hospital, Denville, New Jersey

## Abstract

**Importance:**

Early evidence from studies outside of oncology has suggested that cannabidiol (CBD) may have anxiolytic effects without neuropsychiatric risks. An understanding of oral CBD in patients with cancer-related anxiety is urgently needed.

**Objective:**

To determine whether a single 400-mg oral dose of a US Food and Drug Administration–approved CBD improves clinical anxiety in an oncologic population.

**Design, Setting, and Participants:**

This phase II, double-masked, placebo-controlled, randomized clinical trial was performed at the Dana-Farber Cancer Institute’s Breast Oncology Center from November 2, 2021, through March 1, 2023. Women aged 18 years or older with advanced breast cancer and baseline clinical anxiety were included.

**Interventions:**

Patients were randomized 1:1 to receive oral CBD, 400 mg, vs placebo within 48 hours before a scan assessing tumor burden.

**Main Outcomes and Measures:**

The primary end point was a between-arm comparison of change scores on the afraid subscale of the Visual Analog Mood Scale (VAMS) before and 2 to 4 hours after study drug ingestion. The VAMS scores were converted to T-scores to facilitate interpretation of mood change (>20 indicates a reliable change, >30 indicates both a reliable and clinically significant change). Exploratory outcomes included between-arm comparisons of anxiety levels 2 to 4 hours after study drug ingestion, between-arm comparisons of change scores on other VAMS subscales, and safety.

**Results:**

Among the 50 participants, 25 were randomized to the placebo arm (mean [range] age, 57 [37-81] years) and 25 were randomized to the CBD arm (mean [range] age, 60 [30-79] years). The primary end point of VAMS afraid subscale change score, although numerically greater in the CBD arm, was not significantly different between arms (mean [SD]: CBD, −19.1 [15.4]; placebo, −15.0 [10.9]; *P* = .37). The secondary outcome directly comparing anxiety levels between arms 2 to 4 hours after study drug ingestion demonstrated significantly lower VAMS afraid T-scores for participants who received CBD compared with those receiving placebo (mean [SD]: CBD, 51.5 [12.8]; placebo, 58.0 [11.6]; *P* = .02). No grade 3 or 4 toxic effects were reported.

**Conclusions and Relevance:**

The findings of this randomized clinical trial show that CBD can be used safely in women with advanced breast cancer and clinical anxiety. Although the study did not meet its primary end point comparing preingestion vs postingestion anxiety change scores between study arms, anxiety levels in the CBD arm were significantly lower 2 to 4 hours after ingestion, suggesting a possible anxiolytic effect and warranting further investigation.

**Trial Registration:**

ClinicalTrials.gov Identifier: NCT04482244

## Key Points

**Question:**

Does cannabidiol (CBD) improve cancer-related anxiety?

**Findings:**

In this phase II, double-masked, randomized clinical trial in 50 women with advanced breast cancer and clinical anxiety receiving CBD or placebo before a scan to assess tumor burden, a between-arm comparison of anxiety change before and after study drug ingestion was negative, even as anxiety levels after ingestion were significantly lower in the CBD vs placebo arm. No grade 3 or 4 toxic effects were reported.

**Meaning:**

The findings did not meet the study’s primary end point; however, oral CBD was safe and resulted in significantly lower anxiety levels, suggesting a possible anxiolytic effect.

## Teaser

This randomized clinical trial investigates whether a single oral dose of cannabidiol improves scan-related anxiety in women with advanced breast cancer.

## Introduction

One in every 4 or 5 adults receiving oncologic care meets the criteria for clinical anxiety.^1,2,3,4^ Few advances in the pharmacologic management of acute anxiety—cancer-related or otherwise—have emerged in the past half century. Benzodiazepines are the standard of care, though their myriad risks include confusion, dementia, hypersomnia, perceptual disturbances, amnesia, addiction, and ataxia, leading to injuries and driving liabilities.^5,6^ Such dangers are particularly concerning in oncology, where patients are frequently frail, older, and taking multiple medications.^5^ Rates of benzodiazepine use among adults with cancer have not been published since the 1970s but at the time were reported to range between 16% and 25%.^7^ Current rates are likely higher as the National Comprehensive Cancer Network now identifies this medication class as playing a role in the management of nausea and emesis.^8^ Given the concerns about benzodiazepine use, safer and more effective alternatives are warranted in patients with cancer.

Early evidence from studies outside of oncology suggests that cannabidiol (CBD), a nonintoxicating, nonaddictive *Cannabis sativa* component, may possess anxiolytic effects without the neuropsychiatric risks of δ-9-tetrahydrocannabinol products, for instance, experience of a high, perceptual disturbances, and ataxia.^6,9,10^ Pilot studies have shown through subjective, physiologic, and functional neuroimaging assessments that CBD is associated with improved reactive anxiety to magnetic resonance imaging scanning and public speaking in participants without mental health diagnoses^9,10,11,12^ and in participants with social phobia.^13,14^ This anxiolytic effect, believed to be mediated through serotonin 1A receptor agonism and activity in limbic and paralimbic structures,^6,9^ seems to occur without confusion and with less sedation than with benzodiazepines.^10,13^ Other clinical and/or preclinical studies suggested that CBD is associated with antipsychotic, sedative, anticonvulsive, antiemetic, analgesic, and antineoplastic outcomes.^15,16,17,18^ Furthermore, early evidence has hinted that long-term CBD consumption may be safe in oncology settings.^19^ For instance, a double-masked placebo-controlled trial of a median CBD dose of 400 mg/d over 2 weeks in palliative care patients with advanced cancer led to no grade 3 or 4 toxic effects. However, not enough CBD clinical trial research in oncology populations has been completed to allow for evidence-based clinical recommendations around its utility.^20^ As CBD is the only current US Food and Drug Administration (FDA)–approved herbal cannabinoid product and may present a more benign alternative to benzodiazepines in oncology settings, randomized clinical trials of CBD in adults with cancer with an anxiety primary end point are urgently necessary.

Scan-related anxiety is an elegant, discrete model for oncologic anxiety, encompassing fears pertaining to discomforts associated with the scan itself, of cancer progression, and of the unknown.^21^ Its prevalence among adults with cancer ranges from 23% to 81%.^21^ Most studies that have measured the natural history of scan-related anxiety suggested that the phenomenon peaks prior to the scan rather than after.^21^ Herein, we investigate the role of a single dose of oral CBD, 400 mg, in managing anxiety within the 48 hours preceding a scan to assess tumor burden in an oncology population.

## Methods

This single-institution, phase II, double-masked, placebo-controlled randomized clinical trial evaluated the preliminary efficacy and safety of a single dose of oral pharmaceutical-grade CBD, 400 mg, in treating prescan anxiety in women with breast cancer.^6^ The trial protocol is provided in Supplement 1. Study procedures were approved by the Dana-Farber/Harvard Cancer Center Institutional Review Board. Investigators obtained written informed consent from every participant. This study followed the Consolidated Standards of Reporting Trials (CONSORT) reporting guideline for randomized clinical trials.

Participants were stratified by baseline anxiety levels and randomized 1:1 to receive study drug within 48 hours before a computed tomography (CT) or nuclear medicine positron emission tomography (NM PET) scan to assess tumor burden. We hypothesized that oral CBD, 400 mg, compared with placebo would lead to greater scan-related anxiety reductions (primary end point). Exploratory outcomes included between-arm comparisons of anxiety levels 2 to 4 hours after study drug ingestion, safety, between-arm comparisons of change scores on Visual Analog Mood Scale (VAMS) subscales, and feasibility of study procedures.

### Study Drug

The study drug was an FDA-approved CBD oil (Epidiolex; Jazz Pharmaceuticals) with a time to maximum plasma concentration of 2.5 to 5.0 hours.^22^ A 400-mg dose was selected based on a known 300- to 600-mg anxiolytic band.^6,10,12^ Dana-Farber Cancer Institute’s Research Pharmacy compounded a placebo of karo syrup and strawberry flavoring, matching both the appearance and delivery system (amber, safety-capped 5-mL syringe, sans needle) of the active agent.

### Participants

Women included in the study were 18 years or older with advanced breast cancer defined as evidence of metastatic spread, a Karnofsky performance status of at least 60%, total bilirubin 2 times or less the upper limit of normal, aspartate aminotransferase and alanine aminotransferase 3 times or less the institutional upper limit of normal, baseline anxiety (Generalized Anxiety Disorder-7 [GAD-7] score ≥5), self-reported scan-related anxiety, menopausal status or negative pregnancy test result, ability to speak English, a scheduled CT or NM PET scan, and willingness to avoid cannabinoids within 24 hours and benzodiazepines within 8 hours of study drug administration. Women were excluded if they were allergic to the study drug ingredients; had uncontrolled mental or physical illness or a social situation that impeded participation; were prescribed antiretrovirals, valproic acid, or clobazam; had hepatocellular carcinoma; or were taking part in another clinical drug trial.

### Study Tools

Baseline data collection to allow characterization of the study population included face-valid, self-reported demographics (eg, race [Asian, Black or African American, Native Hawaiian or Pacific Islander, White, multiracial, declined to answer] and ethnicity [Hispanic or Latina, non-Hispanic or Latina], age, marital status) and cannabis history (eg, prior cannabis consumption, reasons for use). The GAD-7, a validated measure of anxiety severity, was administered at baseline and prior to study drug administration.^23^ It operates on a 4-point Likert scale from not at all to nearly every day. A score of 5 or higher on GAD-7 indicates at least mild clinical anxiety.

The VAMS is a validated inventory assessing the following mood states in the moment: afraid, confused, sad, angry, energetic, tired, happy, and tense. Its lack of a recall time frame facilitates tracking of mood states over time as well as monitoring of treatment response. Subscales are measured from 0 to 100 mm and converted to a T-score, a linear transformation of a raw score calculated to have a mean of 50 and an SD of 10.^24,25^ The VAMS professional manual provides researchers with a T-score conversion table based on sex and age bracket (18-54 years and 55-94 years). According to VAMS scoring criteria, pre- and posttest scores on individual items that differ by more than 20 are interpreted as a reliable change in mood, and those differing by more than 30 are interpreted as both a reliable and clinically significant change in mood.^24^ The majority of studies assessing the anxiolytic potential of CBD have used the VAMS.^6^ The Patient-Reported Outcomes version of the Common Terminology Criteria for Adverse Events (PRO-CTCAE) is an adverse events severity grading system ranging from 1 to 5.^26^ Adverse events, including excessive somnolence, hepatotoxicity, seizure, suicidal ideation, nausea, or vomiting were assessed. The following collected measures will be analyzed later and reported in subsequent articles: Patient Health Questionnaire^27^; European Organization for Research and Treatment of Cancer Quality of Life Questionaire-C30 nausea subscale; and Numeric Pain Rating Scale.

### Outcome Measures

The primary outcome measure was change score on the VAMS afraid subscale immediately before and 2 to 4 hours following study drug ingestion. Secondary outcomes included change scores (over the same period) on other VAMS subscales and safety as defined by adverse events. We also assessed feasibility of adherence to the study protocol, specifically surrounding drug delivery and consumption.

### Random Assignment

Central registrars randomized participants 1:1 using permutated blocks within a strata of baseline GAD-7 anxiety scores (5-14, mild to moderate; 15-21, severe). The registrars then informed research pharmacists who dispensed the study drug according to the randomization schedule. Participants, caregivers, investigators, and clinical staff remained masked to study assignment until trial completion.

### Study Procedures

Through Dana-Farber’s Breast Oncology Center, study staff (M.M.N., N.A., S.J., C.V., and I.M.B.) screened medical records between November 2, 2021, and March 1, 2023, and obtained physician approval prior to contacting potential participants. Interested women were consented and completed the survey and laboratory assessments necessary to determine eligibility. Survey assessments included demographics, cannabis history, the validated GAD-7 scale, and history of scan-related anxiety. Eligible women were randomized 1:1 to receive oral CBD, 400 mg, vs placebo. The research pharmacy prepared the study drug on demand, and the research team delivered it to participants within 24 hours of the treatment day. Researchers instructed participants to avoid high-calorie or high-fat meals for 1 hour before and after study drug ingestion. Because study procedures took place during the height of the COVID-19 pandemic, dosing typically occurred in a participant’s home with a researcher present by telephone or videoconferencing. On the treatment day, which occurred within 48 hours of a scheduled CT or NM PET scan, participants completed the VAMS and PRO-CTCAE before ingesting the study drug. Two to 4 hours after ingestion, participants were reassessed with the VAMS and PRO-CTCAE. Approximately 1 week after ingestion, participants took part in a brief semistructured telephone interview assessing satisfaction with the study drug and were paid $50 for participating.

### Statistical Analysis

Anticipating minimal change in the placebo arm, a change score of 20T in the CBD arm was targeted based on pre- and posttest findings in nononcologic settings that 20T indicates a reliable change in mood.^24^ A sample size of 45 was calculated to detect differences between groups with an SD of 20, a power of 80%, and a 2-sided type I error of 0.05. The initial statistical plan was based on a 2:1 double-masked randomization scheme (30 participants receiving CBD and 15 placebo). Allowing for 10% attrition, 50 total participants were targeted. The central registrar inadvertently set the randomization scheme as 1:1, resulting in increased power (89%) to detect the above difference between groups.

Normally distributed variables are described by their mean and SD or, for hypothesis tests, mean and 95% CIs. Variables that were nonnormally distributed are described by their median and range. Categorical variables are reported as counts and percentages. Outcomes comparing before and after drug ingestion were calculated as the value 2 to 4 hours following study drug administration minus the baseline value. All nonnormally distributed variables were compared using Wilcoxon rank sum test and categorical variables with Pearson χ^2^ or Fisher exact tests. All analyses are 2-sided based on a 5% significance level. As defined by the primary protocol (Supplement 1), the afraid subscale difference in T-scores was compared by nonparametric rank sum test.

Sensitivity analyses were performed using parametric *t* tests, linear regression, and analysis of covariance models. For feasibility of the study protocol, we calculated the proportion of study participants who were enrolled and successfully received the study drug. We assessed those who were able to take the study drug and complete follow-up measures. Analyses were conducted using Stata, version 18 (StataCorp LLC).

## Results

Among the 50 participants included, 25 were randomized to the placebo arm (mean [range] age, 57 [37-81] years), and 25 were randomized to the CBD arm (mean [range] age, 60 [30-79] years) (Table 1). Forty-three participants (86%) self-reported as White race (compared with 7 [14%] reporting as Asian, Black or African American, Native Hawaiian or Pacific Islander, or multiracial or declining to report), and 47 (94%) self-reported as non-Hispanic or Latina ethnicity (compared with 3 [6%] reporting as Hispanic or Latina ethnicity). Twelve participants (24%) were not college graduates, 15 (30%) worked full or part time, 32 (64%) were married, 44 (88%) scored in the mild to moderate range for clinical anxiety, 19 (38%) reported using cannabis, 25 (50%) reported using benzodiazepines, 49 (98%) had metastatic breast cancer, and 1 (2%) had lymphatic spread with micrometastases. Thirty-three participants (66%) were scheduled to undergo a CT scan of the chest, abdomen, and/or pelvis and 14 (28%), an NM PET/CT (skull base to midthigh). A mean (SD) of 2.2 (0.4) hours transpired from prestudy drug ingestion to anxiety measurement, and a mean (SD) of 1.4 (0.7) days elapsed from drug ingestion to scheduled scan. No clinically meaningful imbalances between groups were observed. Because only 6 participants (12%) scored in the severe range on the GAD-7, within-strata analyses were not feasible.

**Table 1.** Demographics and Disease Characteristics

Characteristic	No. of patients (%)		
	Placebo (n = 25)	Cannabidiol (n = 25)	Total (N = 50)
Age, mean (range), y	57 (37-81)	60 (30-79)	58 (30-81)
Race			
Asian	1 (4)	0	1 (2)
Black or African American	1 (4)	0	1 (2)
Native Hawaiian or Pacific Islander	1 (4)	0	1 (2)
White	19 (76)	24 (96)	43 (86)
Multiracial	2 (8)	1 (4)	3 (6)
Declined	1 (4)	0	1 (2)
Ethnicity			
Hispanic or Latina	3 (12)	0	3 (6)
Non-Hispanic or Latina	22 (88)	25 (100)	47 (94)
Education^a^			
Grade school	0	1 (4)	1 (2)
High school	2 (8)	3 (13)	5 (10)
Some college	5 (20)	1 (4)	6 (12)
College	7 (28)	12 (50)	19 (39)
Postgraduate degree	11 (44)	7 (29)	18 (37)
Employment status^b^			
Retired	6 (25)	10 (40)	16 (33)
Working full or part time	8 (33)	7 (28)	15 (31)
Receiving disability	5 (21)	3 (12)	8 (16)
Other^c^	5 (21)	5 (20)	10 (20)
Marital status			
Single	6 (24)	4 (16)	10 (20)
Married	16 (64)	16 (64)	32 (64)
Other^d^	3 (38)	5 (63)	8 (16)
Baseline anxiety			
Mild to moderate	22 (88)	22 (88)	44 (88)
Severe	3 (12)	3 (12)	6 (12)
Scan type			
CT of chest, abdomen, and/or pelvis	14 (56)	19 (76)	33 (66)
NM PET/CT skull base to midthighs	9 (36)	5 (20)	14 (28)
CT other (abdomen, midthighs)	2 (8)	1 (4)	3 (0.06)
Benzodiazepine use			
Yes	13 (52)	12 (48)	25 (50)
No	12 (48)	13 (52)	25 (50)
Cannabis consumption			
Yes	9 (36)	10 (40)	19 (38)
No	16 (64)	15 (60)	31 (62)
Stage at initial diagnosis			
DCIS	0	3 (12)	3 (6)
I	5 (20)	1 (4)	6 (12)
II	7 (28)	10 (40)	17 (34)
III	5 (20)	4 (16)	9 (18)
IV	8 (32)	7 (28)	15 (30)
Subtype			
*ERRB2* (formerly *HER2* or *HER2*/neu) not determined	1 (4)	0	1 (2)
*ERRB2* positive/HR positive	1 (4)	5 (20)	6 (12)
*ERRB2* positive/HR negative	1 (4)	0	1 (2)
*ERRB2* negative/HR positive	22 (88)	19 (76)	41 (82)
TNBC	0	1 (4)	1 (2)
Disease-free interval			
De novo MBC presentation	8 (32)	7 (28)	15 (30)
Early stage with lymphatic spread	1 (4)	0	1 (2)
Relapsed in ≤2 y	3 (12)	2 (8)	5 (10)
Relapsed in >2 y	13 (52)	16 (64)	29 (58)
Time from metastatic disease to scan, mean (SD), d	3.3 (2.6)	3.7 (2.7)	3.5 (2.6)
Time from drug ingestion to scan, mean (SD), d	1.4 (0.6)	1.5 (0.9)	1.4 (0.7)
Time from drug ingestion to anxiety measurement, mean (SD), h	2.3 (0.6)	2.1 (0.9)	1.4 (0.7)

Abbreviations: CT, computed tomography; DCIS, ductal carcinoma in situ; HR, hormone receptor; MBC, metastatic breast cancer; NM PET, nuclear medicine positron emission tomography; TNBC, triple-negative breast cancer.

^a^ Missing data on 1 participant who did not provide an answer as to their education.

^b^ Missing data on 1 participant who did not provide an answer as to their employment.

^c^ Included unemployed and/or a student.

^d^ Included separation or divorce and/or widowed.

### Screening for Eligibility

**Figure 1.**
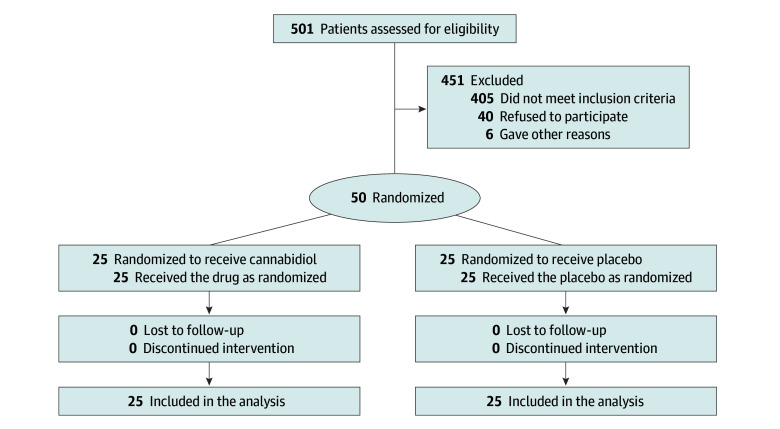
Flow Diagram

Between November 2021 and March 2023, 501 patient medical records were screened (Figure 1). Of 310 patients excluded, 76 were participating in another clinical drug trial, 64 did not respond to outreach, 37 formally declined, and 133 were excluded for other reasons. Of the 191 patients who met preconsent eligibility criteria, 54 did not have a scheduled scan to assess tumor burden, 40 had a scan scheduled too soon to allow for study procedure completion, 27 denied anxiety during the telephone screening, and 3 were excluded for other reasons. Of the 67 potential participants scheduled for consent, 3 lost interest, 3 denied having scan-related anxiety, and 1 did not attend the scheduled consent appointment, leading to 60 patients (90%) consenting to participate. Ten who consented were found ineligible: 5 because they scored below the GAD-7 threshold, 2 because they requested a delay until their next scan (and recruitment had closed), 1 because they were participating in another drug trial, 1 because of a drug interaction risk, and 1 because their scan was too soon to allow for study procedures. The trial concluded after all 50 participants were randomized (25 per arm), consumed the study drug, and completed follow-up assessments for inclusion in the primary analysis.

### Accrual

Of the 200 instances the research team sought oncologic physician permission to approach a potential participant, approvals were granted for 182 (91%) patients. Of 120 introductory telephone conversations conducted with potential participants, 86 (72%) expressed interest.

### Primary End Point

Immediately before study drug ingestion, VAMS afraid subscale T-scores were similar between study arms (mean [SD]: CBD, 70.6 [12.5]; placebo, 73.0 [11.6]; *P* = .60). Two to 4 hours after ingestion, the mean (SD) VAMS afraid T-scores were 51.5 (12.8) in the CBD arm and 58.0 (11.6) in the placebo arm. Change in VAMS afraid T-scores before and after study drug ingestion, although greater in the CBD arm, did not differ significantly between groups (mean [SD]: CBD, −19.1 [15.4]; placebo, −15.0 [10.9]; *P* = .37) (Table 2).

**Table 2.** VAMS Change Scores and Spearman Correlations

VAMS subscale	Mean (SD)			*P* value^a^	Before vs after drug ingestion T-score correlation, *r* (95% CI)
	Placebo	Cannabidiol	Total		
Afraid	−15.0 (10.9)	−19.1 (15.4)	−17.1 (13.4)	.37	0.39 (0.12-0.60)
Confused	−0.1 (4.9)	0.9 (6.7)	0.4 (5.9)	.64	0.72 (0.55-0.83)
Sad	−8.9 (11.4)	−5.8 (11.2)	−7.3 (11.3)	.17	0.65 (0.45-0.78)
Angry	−6.1 (10.5)	−3.6 (8.8)	−4.8 (9.7)	.26	0.62 (0.41-0.76)
Energetic	−2.1 (13.6)	−1.8 (8.7)	−2.0 (11.3)	.84	0.40 (0.14-0.61)
Tired	−3.6 (10.1)	−2.0 (9.8)	−2.8 (9.9)	.35	0.40 (0.14-0.61)
Happy	2.6 (9.3)	3.3 (8.7)	2.9 (8.9)	.65	0.72 (0.60-0.83)
Tense	−10.4 (8.8)	−11.6 (12.6)	−11.0 (10.8)	.68	0.48 (0.23-0.67)

Abbreviation: VAMS, Visual Analog Mood Scale.

^a^ By 2-tailed Wilcoxon rank sum test.

### VAMS Afraid Subscale Exploratory End Points

**Figure 2.**
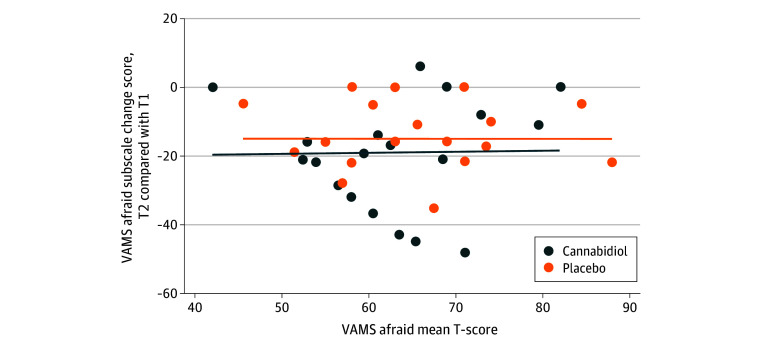
Bland-Altman Plot of Visual Analog Mood Scale (VAMS) Afraid Subscale Change Scores vs Mean VAMS Afraid T-Scores T1 indicates before study drug ingestion; T2, 2 to 4 hours after study drug ingestion.

Compared with participants randomized to the placebo arm, those who received CBD reported significantly lower VAMS afraid scores 2 to 4 hours after ingestion (mean [SD]: CBD, 51.5 [12.8]; placebo, 58.0 [11.6]; *P* = .02) (Table 3). On a Bland-Altman scatter plot, the VAMS afraid scale change scores were directly correlated with prestudy drug ingestion VAMS afraid T-scores (Figure 2). T-scores immediately before study drug ingestion were moderately correlated with those after ingestion (CBD, *r* = 0.30 [95% CI, −0.11 to 0.62]; placebo, *r* = 0.53 [95% CI, 0.17-0.76]) (Table 2).

**Table 3.** VAMS T-Scores 2 to 4 Hours Following Study Drug Ingestion

VAMS subscale	Mean (SD)			*P* value^a^
	Placebo	Cannabidiol	Total	
Afraid	58.0 (11.6)	51.5 (12.8)	54.7 (12.5)	.02
Confused	42.9 (4.2)	43.1 (5.3)	43.0 (4.7)	.35
Sad	48.4 (11.7)	45.4 (11.7)	46.9 (11.7)	.046
Angry	45.8 (8.4)	43.4 (3.3)	44.6 (6.4)	.36
Energetic	38.8 (9.8)	40.2 (10.9)	39.5 (10.3)	.81
Tired	49.8 (9.0)	50.8 (10.3)	50.3 (9.6)	.65
Happy	43.9 (12.6)	46.7 (11.6)	45.3 (12.0)	.64
Tense	50.1 (8.5)	47.9 (12.4)	49.0 (10.6)	.14

Abbreviation: VAMS, Visual Analog Mood Scale.

^a^ By 2-tailed Wilcoxon rank sum test.

### Adverse Events

No grade 3 or 4 PRO-CTCAE toxic effects, including somnolence, hepatotoxicity, seizure, suicidal ideation, nausea, or vomiting, were reported. In the CBD arm, 1 participant (4%) reported grade 1 nausea and 1 (4%) reported grade 2 nausea and pain. All toxic effects were deemed possibly related to the study drug.

### Other VAMS Subscales

No statistically significant differences in change scores were observed for any VAMS subscales (Table 2). Before ingestion, no statistically significant differences between study arms existed for any VAMS subscale. After ingestion, participants in the CBD arm reported statistically lower VAMS sad subscale T-scores compared with those in the placebo arm (Table 3). Correlations between pre- and postingestion VAMS subscale scores ranged from 0.40 (95% CI, 0.14-0.61) for both the VAMS energetic and tired subscales to 0.72 (95% CI, 0.55-0.83) for the VAMS happy and 0.72 (95% CI, 0.60-0.83 for the VAMS confused subscales (Table 2).

## Discussion

Responding to a crucial need for innovative pharmacotherapies for acute oncologic anxiety, this double-masked, placebo-controlled randomized clinical trial is, to our knowledge, the first oncologic CBD study with an anxiety-related primary end point: a between-arms comparison of change in anxiety in women who have advanced breast cancer, baseline anxiety, and a pending scan to assess tumor burden. While the primary end point was not statistically significant, women randomized to the CBD arm reported significantly lower anxiety and dysphoria 2 to 4 hours following study drug ingestion. Furthermore, participants in the CBD arm experienced numerically larger drops in anxiety levels (CBD, −19.1; placebo, −15.0), with a change score of more than 20 considered a reliable change in mood on the VAMS inventory. Importantly, oral CBD 400 mg was safely tolerated by our participants, with no significant adverse events. We believe these signals are sufficiently intriguing to justify continued exploration of CBD as a safe and possibly effective therapy for cancer-related anxiety.

Only 2 prospective clinical trials, including ours, have examined CBD’s effects on cancer-related anxiety. The first, a placebo-controlled CBD trial, demonstrated negative findings for a secondary anxiety end point.^28,29^ Its primary focus was on pansymptom management, and, understandably, the trial design did not enrich for anxiety at baseline or prohibit benzodiazepine use during the study period. Furthermore, the 2 in-the-moment anxiety scores reported in the study were assessed 2 weeks apart. Finally, the trial’s active agent was not pharmaceutical grade. By contrast, our investigation features important design strengths. We used a scan-related model for cancer anxiety, which allowed us to isolate the effects of study drug on a specific anxiety trigger, and prohibited benzodiazepines and other cannabinoids on the treatment day. We used validated scales to measure anxiety, and our measurement time frame was on the order of hours, minimizing confounding due to natural anxiety fluctuations. Importantly, our selection of a fixed-dose, FDA-approved CBD product facilitates reproducibility of this trial design and its expansion to other potential end points. If efficacy were to be demonstrated, the use of an already-approved drug and formulation might facilitate clinicians offering a safe and reliable alternative to conventional medications for acute anxiety.

Our study offers methodological learning. The absence of statistical significance for the primary result may have been due to a modest sample size, the small differences in means between groups at baseline, and relatively low correlation between VAMS afraid subscale scores before and after study drug ingestion. In fact, the degree of correlation between the pre- and poststudy drug ingestion scores was lower for the VAMS afraid subscale than for the other VAMS subscales. We selected anxiety change scores as a primary end point with the assumption that preingestion anxiety levels would serve as statistical controls for postingestion ones; however, empirically, preingestion anxiety levels did not perform as expected. As Figure 2 illustrates, participants with the highest anxiety levels before study drug ingestion showed the largest drops in anxiety following ingestion. While this phenomenon may be due to regression to the mean, it also suggests that the study would have been better served by narrowing inclusion criteria to participants reporting moderate or severe anxiety. The hypothesis that the study’s impact would be reduced by inclusion of individuals with mild anxiety is supported by another cancer-related anxiety intervention trial.^30^

Our study also demonstrated that clinical trials treating anxiety with CBD are attractive to adults with cancer and their oncologists and that the medication is tolerable. Oncologists granted the study team permission to approach the majority of potential study participants, including octogenarians and those with advanced disease, suggesting that these clinicians deemed CBD’s benefits to outweigh its risks. Study procedures occurred during the height of the COVID-19 pandemic when clinical trial recruitment stagnated. Even so, nearly three-quarters of potential participants expressed interest when approached. Furthermore, clinicians proactively recommended patients for the study, participants referred contacts to the study, and patients self-referred. With regard to tolerability, our trial, which used FDA-approved pharmaceutical grade CBD, contributes to a growing body of literature suggesting that 400 mg of oral CBD may be used safely in patients with cancer, including those of advanced age, with advanced disease, with immunocompromise, and/or who are hospitalized.^19^ This finding may provide important learning for other CBD clinical trialists as well as those advising adults with cancer in the clinic.

### Limitations

This study had several limitations. First, it excluded men, so its findings cannot be extrapolated beyond women. Second, the VAMS inventory, while commonly used in CBD studies with anxiety end points, is not validated in adults with cancer, even though it is increasingly used in chronic illness research. Third, time from initial diagnosis, number of scans, current cancer-directed therapy, and not excluding participants taking antidepressants are potential confounders. Fourth, a single dose of CBD was administered, so the potential benefits and risks of repeated dosing were not evaluated. One such risk is that of reversible liver enzyme elevations with long-term, daily CBD dosing of more than 300 mg.^20^ Finally, with an eye to justice and inclusivity, we regret that our study was limited to English speakers. However, a unique design strength was the at-home study procedures, which we believe facilitated participation of a sociodemographically and culturally diverse population.

## Conclusions

This preliminary randomized clinical trial establishes CBD as a treatment for anxiety for patients with breast cancer and has laid groundwork for a larger, more definitive trial to test the efficacy of oral CBD, 400 mg, in decreasing anxiety in this patient population. Data from this study invite completion of a longitudinal trial that incorporates anxiety score trajectories on repeated in-the-moment measures, use of potential wearable devices to objectively measure anxiety phenomena, inclusion of participants with moderate to severe anxiety levels, and use of the Patient Global Impressions Scale. Such a study may allow the scientific community to truly assess whether oral CBD, which we show can be used safely in a medically ill population, benefits adults with cancer and acute clinical anxiety.

## Notes

Supplement 1.
Trial Protocol

Supplement 2.
Data Sharing Statement
