# Supplementary material for: Cannabidiol for Scan-Related Anxiety in Women With Advanced Breast Cancer: A Randomized Clinical Trial
Source: JAMA Netw Open. 2024 Dec 16;7(12):e2450391. doi: 10.1001/jamanetworkopen.2024.50391 (PMC11650394; doi:10.1001/jamanetworkopen.2024.50391)
Supplement: Supplement 1. — Trial Protocol [file jamanetwopen-e2450391-s001.pdf]

DF/HCC BIOMEDICAL PROTOCOL TEMPLATE
Version Date:
October 13, 2022

NCI Protocol #: Not Applicable

DF/HCC Protocol #: 19-886

TITLE: Randomized, Double-Blind Phase II Study of a Single Dose of Cannabidiol (CBD) Versus Placebo for Acute Anticipatory Anxiety in Advanced Breast Cancer

Coordinating Center: Dana-Farber Cancer Institute

***Principal Investigator (PI):** Ilana Braun, MD
Dana-Farber Cancer Institute (DFCI)
ibraun@partners.org

Other Investigators: Peter Chai, MD
Dana-Farber Cancer Institute
pchai@partners.org

William Pirl, MD
Dana-Farber Cancer Institute
William_pirl@dfci.harvard.edu

Manan Nayak, PhD
Dana-Farber Cancer Institute
Manan_nayak@dfci.harvard.edu

Stephanie Tung, MD
Dana-Farber Cancer Institute
stephanie_tung@dfci.harvard.edu

Statistician: Paul Catalano, Sc.D
Dana-Farber Cancer Institute
pcata@jimmy.harvard.edu

NCI-Supplied Agent(s): *None*
Other Agent(s): cannabidiol **(**Epidiolex), commercial, GW Pharmaceuticals
Study Exempt from IND Requirements per 21 CFR 312.2(b).

Protocol Type / Version # / Version Date: Original, Version 1.9,
October 13, 2022

SCHEMA

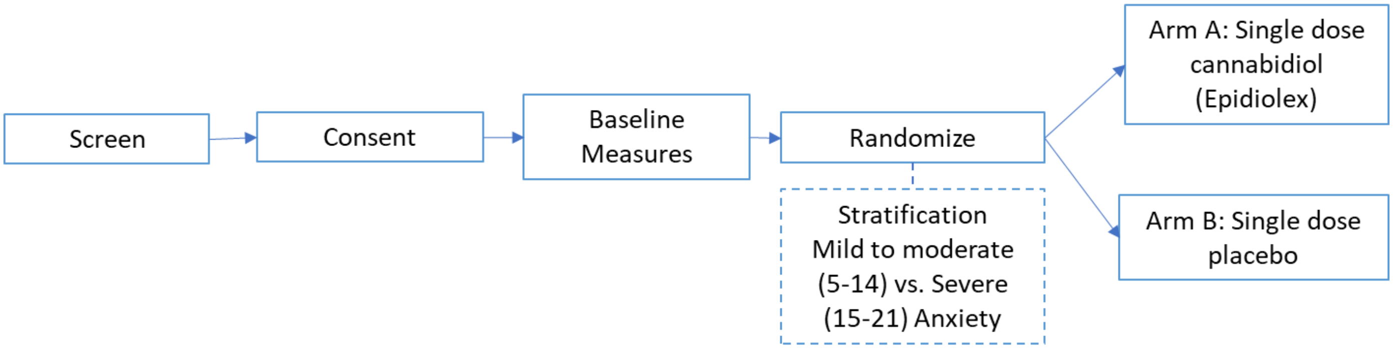
Screen
Consent
Baseline Measures
Randomize
Stratification
Mild to moderate
(5-14) vs. Severe
(15-21) Anxiety
Arm A: Single dose cannabidiol (Epidiolex)
Arm B: Single dose placebo

TABLE OF CONTENTS

SCHEMA 3

1. OBJECTIVES 6
   - 1.1 Study Design 6
   - 1.1 Primary Objectives 6
   - 1.2 Secondary Objectives 6

2. BACKGROUND 6
   - 2.1 Study Disease(s) 6
   - 2.2 IND Agent 6
   - 2.3 Rationale 8
   - 2.4 Correlative Studies Background 9

3. PARTICIPANT SELECTION 9
   - 3.1 Eligibility Criteria 9
   - 3.2 Exclusion Criteria 10
   - 3.3 Inclusion of Women and Minorities 11

4 REGISTRATION And Randomization PROCEDURES 11
   - 4.1 General Guidelines for DF/HCC Institutions 11
   - 4.2 Registration Process for DF/HCC Institutions 11
   - 4.3 General Guidelines for Other Investigative Sites 11
   - 4.4 Registration Process for Other Investigative Sites 11

5. TREATMENT PLAN 12
   - 5.1. Treatment Regimen 12
   - 5.2. Pre-Treatment Criteria 15
   - 5.3. Agent Administration 16
   - 5.4. General Concomitant Medication and Supportive Care Guidelines 17
   - 5.5. Criteria for Taking a Participant Off Protocol Therapy 17
   - 5.6. Duration of Follow Up 17
   - 5.7. Criteria for Taking a Participant Off Study 17

6. DOSING DELAYS/DOSE MODIFICATIONS 18

7. ADVERSE EVENTS: LIST AND REPORTING REQUIREMENTS 18
   - 7.1. Expected Toxicities 19
   - 7.2. Adverse Event Characteristics 20
   - 7.3. Adverse Event Reporting 20
   - 7.4. Reporting to the Food and Drug Administration (FDA) 21
   - 7.5. Reporting to Hospital Risk Management 21
   - 7.6. Routine Adverse Event Reporting 21

8. PHARMACEUTICAL INFORMATION 22

8.1. Cannabidiol (Epidiolex)....22
8.2. *Placebo* 23

9. STUDY CALENDAR 26

10. DATA REPORTING / REGULATORY REQUIREMENTS....27
10.1. Data Reporting....27
10.2. Data Safety Monitoring....27

11. STATISTICAL CONSIDERATIONS....28
11.1. Study Design/Endpoints....28
11.2. Sample Size, Accrual Rate and Study Duration 28
11.3. Stratification Factors....29
11.4. Interim Monitoring Plan 29
11.5. Analysis of Primary Endpoints....30
11.6. Analysis of Secondary Endpoints....30
11.7. Reporting and Exclusions 30

12. PUBLICATION PLAN 30

REFERENCES 31

1. OBJECTIVES

To test the preliminary efficacy of a single dose of oral cannabidiol (CBD) in reducing anticipatory anxiety in patients with advanced cancer poised to undergo computed tomography (CT) scan or positron emission tomography (PET) to assess tumor burden.

Hypotheses
A single dose of oral cannabidiol is superior to placebo in reducing anticipatory anxiety in advanced stage cancer patients with pre-existing anxiety poised to undergo CT or PET to assess tumor burden.

1.1 Study Design

Randomized, double-blind, placebo-controlled trial of a single dose of CBD for anticipatory anxiety in advanced cancer patients.

1.1 Primary Objectives

In patients with advanced cancer poised to undergo CT or PET to assess tumor burden, to evaluate the preliminary efficacy of oral CBD, as compared to placebo, in reducing anticipatory anxiety as measured by the "Afraid" subscale of the Visual Analog Mood Scale (VAMS).

1.2 Secondary Objectives

To assess, using the Patient-Reported Outcomes version of the Common Terminology Criteria for Adverse Events (PRO-CTCAE™) and a qualitative assessment, patient–reported side effects and acceptability of taking oral CBD (versus placebo) for managing anticipatory anxiety.

To evaluate whether CBD is superior to placebo in improving mood (as measured by VAMS subscales), nausea (as measured by the European Organisation for Research and Treatment of Cancer core quality of life questionnaire-nausea subscale) and pain levels (as measured by the Numeric Pain Rating Scale [NPRS]) 3(+/-1) hours after study agent administration in patients with advanced cancer.

2. BACKGROUND

2.1 Study Disease(s)

Advanced breast cancer (stage IV or metastatic)

2.2 IND Agent

The IND agent, cannabidiol (Epidiolex), is manufactured by GW Pharmaceuticals and is Federal Drug Administration (FDA)-approved for management of seizures associated with some form of

pediatric epilepsy in patients 2-years of age or older.[1] Cannabidiol (Epidiolex) is a solution intended for oral consumption that contains, in addition to CBD (100 mg/mL), the following inactive ingredients: sesame seed oil, sucralose, alcohol and strawberry flavor.

Cannabidiol ($C_{21}H_{30}O_2$; molecular weight=314.46) is a non-intoxicating, non-addictive cannabis ingredient. A single clinical trial has been carried out in an oncology population. In this single-arm phase II trial targeting graft-versus-host disease prophylaxis, CBD was found to be safe for use (and of promising effectiveness) in patients undergoing allogeneic hematopoietic cell transplantation[2]. Preclinical and clinical studies suggest that CBD possesses a variety of other therapeutic benefits with potential relevance to cancer patients. These include anxiolytic, antipsychotic, sedative, anticonvulsive, antiemetic, analgesic, and antitumor effects [3-6]. Zeroing in on evidence regarding anxiolysis, using a cross-over model, Crippa et. al. demonstrated through subjective report, physiological parameter testing, and functional neuroimaging that CBD significantly ameliorates reactive anxiety in healthy normals (2004; N=10; anxiety provoking stimulus: MRI), those with generalized social anxiety disorder (2011; N=10), and those with social phobia N= 30[7]. Although the agent's mechanism of action has yet to be fully elucidated, $5HT_{1A}$ receptor agonism is postulated to mediate such anti-anxiety effects[5,7].

In all human studies (including these), CBD is well-tolerated and, despite existing in the same plant as delta-9-tetrahydrocannabinol, manifests divergent and sometimes opposing effects[3,5]. For instance, in sharp contrast to delta-9-tetrahydrocanninol, CBD does not trigger abuse, dependence or withdrawal in preclinical studies. According to Epidiolex's package insert, its most common adverse reactions-- based on chronic twice-daily dosing-- include somnolence, decreased appetite, diarrhea, fatigue, malaise, asthenia, rash, insomnia, sleep disorder, poor quality sleep, infection, and dose-related transaminase elevations. When they occur, transaminase elevations typically present in the first two months of use of the drug, and mainly in patients on concomitant valproate. In a third of patients, the transaminase elevations improve without intervention. In two-thirds, the transaminase elevations respond to reduction or discontinuation of CBD, valproate, or both. Like all antiepileptic drugs, CBD carries a warning to monitor for suicidal behavior; however, clinical data from the pediatric trials does not seem to demonstrate increase in suicidal behavior compared to patients on placebo. Epidiolex's recent FDA approval, coupled with the United States Drug Enforcement Administration's (DEA) descheduling, has openned doors to clinical trials of CBD in oncology.

Preclinical models have demonstrated CBD to have a U-shaped dose response curve with respect to anxiety, with higher doses (20 or100 mg/kg) ineffective and lower dose CBD (10 mg/kg) effective[5]. In humans, 300-600 mg doses were found to have anxiolytic effects[8]. According to the Epidiolex package insert, CBD "demonstrate[s] an increase in exposure that [is] less than dose-proportional over the range of 5 to 20 mg/kg/day." Cannabidiol has a time to maximum plasma concentration (Tmax) of 2.5-5 hours at steady state. The effects of food are significant: Taking CBD in the setting of a caloric, high-fat meal increases the C_{max} 5-fold and the area under the curve (AUC) 4-fold. It also reduces the total variability, compared to the fasting state, in healthy volunteers. Cannabidiol's volume of distribution in healthy volunteers is 20,963 L to 42,849 L. Protein binding of the drug and its metabolites is >94% in vitro. The half-life of CBD in the plasma is 56-61 hours after a week of twice-daily dosing in healthy volunteers. Plasma clearance following a single 1500 mg CBD dose is 1111 L/h. Cannabidiol is metabolized by

CYP3A4 and CYP2C19. In the presence of moderate or strong inhibitors of these enzymes, dose reductions of CBD should be considered; in the presence of strong inducers, dose increases of CBD should be considered. Cannabidiol impedes metabolism of substrates of UGT1A9, UGT2B7, CYP2C8, CYP2C9, CYP2C19, and to an extent, CYP1A2 and CYP2B6. The somnolence and sedation experienced with CBD may be compounded by other central nervous system depressants such as alcohol or benzodiazepines. Of note, CBD is excreted in the feces with only mild renal clearance.

2.3 Rationale

In the last half century, there have been few advances in the pharmacologic management of acute anxiety. As one example, a panic attack in 2019 is managed as it was in 1969: with a class of medications called benzodiazepines. While effective, benzodiazepines carry hefty risk profiles that include, acutely, cognitive slowing, even frank confusion, hypersomnia, perceptual disturbances, anterograde amnesia, accidental overdose, ataxia and hypotension, leading to falls and other injuries. Long-term benzodiazepine use may result in dependence, abuse, and a withdrawal reaction upon discontinuation.

The lack of pharmacologic innovation in the management of acute anxiety is particularly concerning to oncology, where anxiety is ubiquitous. According to one study, 42% of cancer patients manifest clinical anxiety after cancer diagnosis[9]. Cancer-related concerns are myriad and include health and existential anxieties (for instance, after receipt of alarming test results), psychosocial worries (for instance, around the impact of cancer on occupation and family), and fears regarding procedures (for instance, chemotherapy infusions, surgeries and scans to assess tumor burden). Of note, the anxiety tied to radiographic procedures to assess tumor burden peaks prior to the actual scan (rather than during or after).[10,11] While management strategies include relaxation techniques, psychological treatments such as counseling, and antidepressants, the mainstay of treatment for moderate to severe acute cancer-related anxiety remains benzodiazepines. Although rates of benzodiazepine use among cancer patients have not been published in the last three decades, previously published rates range from 16-25% [12] We suspect that current rates are higher, first because prescription benzodiazepine use is on the rise in the United States[13] and, second, because the National Comprehensive Cancer Network now identifies benzodiazepines among the chief management strategies for anticipatory nausea and vomiting with chemotherapy[14]. Of note, the DFCI website prominently encourages use of benzodiazepines prior to radiographic imaging[15].

Common, however, is not necessarily good. The risks attending benzodiazepine use can be magnified in cancer patients who are more likely to be frail and elderly. In addition, benzodiazepine use prior to an oncology appointment in which important medical information is to be transmitted may impair patient comprehension and decision-making ability. In the general public and, especially, in cancer populations, safer, effective alternatives to benzodiazepines are warranted.

Cannabidiol, a non-intoxicating, non-addictive cannabis ingredient, may confer similar benefits to benzodiazepines with fewer risks[7,16]. Preclinical and clinical studies suggest that CBD possesses anxiolytic, antipsychotic, sedative, anticonvulsive, antiemetic, anti-inflammatory,

analgesic, and antitumor effects [3-6]. For instance, using a cross-over model, Crippa et. al. demonstrated through subjective report, physiological parameter testing, and functional neuroimaging that CBD significantly ameliorates reactive anxiety in healthy normals (2004; N=10; anxiety provoking stimulus: MRI), those with generalized social anxiety disorder (2011; n=10), and those with social phobia;[7] n=30). Although the biochemical mechanism of cannabidiol has not yet been fully elucidated, $5HT_{1A}$ receptors in limbic and paralimbic areas are postulated to mediate is anxiolytic effects[7].

In all human studies (including these), CBD was well-tolerated and, despite existing in the same plant as tetrahydrocannabinol, seems to have different and sometimes opposing effects to it [3,5]. Administered acutely across a wide variety of oral (15 mg to 600 mg), intravenous (5 to 30 mg) and inhaled (0.15 mg/kg) doses, CBD produced no notable emotional, cognitive, psychomotor or vital sign side-effects[7]. Similarly, neither chronic administration up to 300 mg orally for 4.5 months (n=15) nor, in a case report, 1500 mg/day for four weeks produced notable side-effects[17,18]. In the case of the former, neurological and physical examinations, blood and urine analyses, electrocardiograms and electroencephalograms were performed at regular intervals. In fact, the United States Food and Drug Administration (FDA) recently approved pharmaceutical-grade CBD manufactured by GW Pharmaceuticals under the trade name Epidiolex for management of forms of pediatric epilepsy. This approval, coupled with the United States Drug Enforcement Agency (DEA) descheduling of it has openned the door more widely to clinical trials of CBD in oncology.

In this study we hope to test the preliminary efficacy of a single dose of oral cannabidiol (CBD) in reducing anticipatory anxiety in patients with advanced breast cancer. The inciting stressor in the study is anticipation of radiographic assessment of tumor burden. Research indicates that, in cancer patients undergoing positron emission tomography/computed tomography (PET/CT) scans, prescan anxiety is considerably elevated above post-precedure levels[19] This finding is likely due to concerns about undergoing the procedure itself coupled with concerns about the prognostic results it may herrald[19]. Of note, no differences in anxiety amplitude were noted between patients who were naïve to the procedure and those who had undergone PET/CT previously[19].

2.4 Correlative Studies Background

N/A

3. PARTICIPANT SELECTION

3.1 Eligibility Criteria

3.1.1 Diagnosis of Stage IV or metastatic breast cancer

3.1.2 Age ≥18 years.

3.1.3 Karnofsky ≥60%.

3.1.4 Participants must have adequate organ and marrow function at baseline as defined below:

- total bilirubin ≤2 times institutional upper limit of normal (ULN)
- AST(SGOT)/ALT(SGPT) ≤3 × institutional ULN

3.1.5 Baseline at least mild anxiety as measured by GAD-7 ≥5

3.1.6 Experience of at least mild anxiety typically tied to anticipation of oncologic scans (as measured by a prescreen survey item)

3.1.7 Computed tomography or positron emission tomography (PET) to assess tumor burden scheduled for within 48 hours of study drug administration

3.1.8 No cannabis, delta-9-tetrahydrocannabinol or cannabidiol use within 24-hours of study drug administration.

3.1.9 No benzodiazepine consumption within 8 hours of study drug administration (e.g., nighttime benzodiazepine use permissible)

3.1.10 No driving for 12 hours following study drug administration.

3.1.11 English proficiency

3.1.12 The effects of cannabidiol (Epidiolex) on the developing human fetus are unknown. For this reason and because cannabis is known to be teratogenic, women of child-bearing potential must not be pregnant when entering the study. The study team will encourage women of child-bearing age to use adequate contraception (hormonal or barrier method of birth control; abstinence) prior to study entry and for the duration of study participation. Should a woman become pregnant or suspect she is pregnant while she is participating in this study, she should inform her treating physician immediately. Women must agree to use adequate contraception prior to the study, for the duration of study participation, and for 1 week after cannabidiol (Epidiolex) consumption. Women either age ≥ 54 years, or documented to be in menopause, or status post hysterectomy will not be required to obtain bHCG.

3.1.13 Ability to understand and the willingness to sign a written informed consent document.

3.2 Exclusion Criteria

3.2.1 History of allergic reactions attributed to compounds of similar chemical or biologic composition to cannabidiol (Epidiolex) or placebo (which contains sesame, corn and gluten)

3.2.2 Current clobazam or valproic acid use

3.2.3 Current uncontrolled illness, for instance sepsis, symptomatic congestive heart failure, unstable angina pectoris, cardiac arrhythmia

3.2.4 Current use of antiretroviral therapy

3.2.5 Participants with psychiatric illness or social situations that would limit compliance with study requirements

3.2.6 Current hepatocellular carcinoma or documented history of difficult to control diabetes

3.2.7 Active participation in a clinical drug trial

3.3 Inclusion of Women and Minorities

Women of all races and ethnic groups are eligible for this trial. Men will be excluded.

4 REGISTRATION AND RANDOMIZATION PROCEDURES

4.1 General Guidelines for DF/HCC Institutions

Institutions will register eligible participants in the Clinical Trials Management System (CTMS) OnCore. Registrations must occur prior to the initiation of any protocol-specific therapy or intervention. Any participant not registered to the protocol before protocol-specific therapy or intervention begins will be considered ineligible and registration will be denied.

An investigator will confirm eligibility criteria and a member of the study team will complete the protocol-specific eligibility checklist. The randomization in this trial will be stratified on baseline anxiety level: Mild to moderate (5-14) versus Severe (15-21).

Following registration, participants may begin protocol-specific therapy and/or intervention. Issues that would cause treatment delays should be discussed with the Overall Principal Investigator (PI). If the subject does not receive protocol therapy following registration, the subject must be taken off study in the CTMS (OnCore) with an appropriate date and reason entered.

4.2 Registration Process for DF/HCC Institutions

Applicable DF/HCC policy (REGIST-101) must be followed and adobe e-sign for consent

4.3 General Guidelines for Other Investigative Sites

Not Applicable

4.4 Registration Process for Other Investigative Sites

Not Applicable

5. TREATMENT PLAN

5.1. Treatment Regimen

5.1.1 Enrolling sites and study entry

A sample of 200 participants who are scheduled undergo computed tomography (CT) scan or positron emission tomography (PET) to assess tumor burden will be recruited to a randomized control study in which they will receive a single dose of cannabidiol (Epidiolex) versus placebo. Accrued patients will continue on the study and will be randomized only if they meet the eligibility criteria at baseline (described below). Consenting and accrual will stop early if 50 patients meeting eligibility (final sample needed for the study) are randomized and enrolled on to the study.

A study team member will screen potential participants using the daily appointment logs of the DFCI Breast Oncology Center and the DFCI Department of Imaging. The study will also accept referrals from the Breast Oncology Program. We will also obtain a list of participants on 09-204, to aid in our recruitment activity. We will present the study at patient forums; post study flyers in waiting rooms including virtual screens; post the study to DFCI websites (e.g., YAP@DFCI)distribute flyers at local cannabis dispensaries and cannabis certifiers to inform participants about the study (Appendix A: Study Letter & Recruitment Flyer). We will obtain a HIPAA waiver to enable medical record screening of potential participants. Once potential participants are identified, we will notify the primary oncologist of their patient's eligibility via pager, email or in person. Providers need only contact the study team if they do not wish their patient to participate in the study.

Study staff will approach the patient in clinic or schedule a virtual visit with the potential participants to introduce the study (Appendix A: Study Letter & Recruitment Flyer). They will assess eligibility and give potential participants time to ask questions and consider study participation. A licensed physician will answer questions and obtain consent from eligible participants (either in-person or remotely using Adobe eSign).

Once inclusion criteria have been met and exclusions ruled out, study staff will follow the registering processes described in Section 4.0. Participants will be randomized in a 2:1 fashion to CBD or placebo using permuted blocks within strata and a double-blind procedure will be followed. Randomization will be completed by the Office of Data Quality (ODQ). The eligibility checklist(s) and all pages of the consent form(s) will be faxed to the ODQ at 617-632-2295. The ODQ will (a) review the eligibility checklist, (b) register the participant on the protocol, and (c) randomize the participant.

All survey assessments will be completed either in-person or remotely (either through RedCap, phone, text messaging or by mail).

<u>Baseline:</u> Following consent, if necessary (i.e., no such values exist in the chart within the last 6 weeks +/- 7 days), the patient will complete laboratory assessments, as well as a series of

surveys. If during the course of these assessments, the patient is determined to not meet eligibility criteria, they will be taken off study. Once the study team receives baseline assessment, the participant will be scheduled for Study Timepoint 1, which will take place within 48 hours of the anticipated CT scan or positron emission tomography (PET) to assess tumor burden. Study staff will follow up via phone, email, and/or mail to remind the study participant of the scheduled study visit.

Treatment Day:

The Treatment Day will take place within 48 hours of anticipated CT scan or positron emission tomography (PET) to assess tumor burden (based on the study participant's radiology appointment schedule). The study team will contact the participant approximately 24 hours before Timepoint 1 to ensure that the participant abstains from cannabis and benzodiazepine uses. Timepoint 1 (Pre-drug Administration Visit) will be completed prior to study drug administration. Observation for the drug administration is noted in section 5.3. Timepoint 2 (Post-drug Administration) will take place approximately 3(+/-1) hours following study drug administration. In total the treatment day inclusive of timepoint 1 and timepoint 2 will be approximately 8 hours(+/-1).

Timepoint 1 (Pre-drug Administration): Regardless of randomization arm, all participants will complete a set of questionnaires. The study team will review questionnaire responses assessing cannabis and benzodiazepine uses (e.g. section 3.1.8 & 3.1.9 of the eligibility criteria). If a participant indicates they have consumed either within the prohibited timeframe, the study team will attempt to delay study drug administration in order to meet eligibility criteria. (Section 5 offers full details on consuming study drug). However, if the study drug administration cannot be delayed, the Treatment Day will be rescheduled to coincide with the next scheduled CT scan or positron emission tomography (PET). See section 8.1.11. for unused study drug destruction.

Timepoint 2 (Post-drug Administration): All participants will complete a set of questionnaires.

Timepoint 3: Study staff will phone the participant within a week +/- 7 days of study drug consumption to conduct a brief, semi-structured audio-recorded interview.

At study completion participants will receive a $50 gift card. They will also receive up to $50 for qualifying study-related travel costs and/or expenses. After all study procedures are completed, the research team will contact participants to inform of which arm they were assigned after unblinding and share any publications that come from this study.

5.1.2 Treatment Regimen

Both arms: A physician researcher will order the trial drug. The research pharmacy will dispense cannabidiol (Epidiolex) versus placebo in a securely capped amber dosing syringe packaged in bubble wrap. The dose will be made available for pick-up or mailed. The Treatment Day will either occur on or off campus. Irrespective of the study arm to which the patient is assigned, the research team will ask study participants to refrain from consuming high-calorie or -fat meals for

one hour before and after study agent consumption. Examples of foods to avoid include those high in butter, cream, oil, cheese, whole milk, mayonnaise, salad dressing, jam, syrup, honey, nuts, as well as soft drinks, juices, sweets or desserts, pastas, breads, crackers, chips and meats. Reported adverse events and potential risks are described in Section 7. Below outline specifies associated with individual arm.

<u>Cannabidiol (Epidiolex) arm:</u> Cannabidiol (Epidiolex) is an FDA-approved commercially-available oral solution that will be self-administered once in a 400 mg (4 mL) dose. The study drug will be consumed within 5 minutes or less. There will be no observation period post dosing. No dose modifications will be necessary. Another dose will not be administered if the participants vomits after dosing.

<u>Placebo arm:</u> The Dana-Farber Research Pharmacy will create the following non-sterile placebo formulation: Karo Light Corn Syrup and FLAVORx strawberry cream flavoring in a ratio 20 ml:7 drops. Karo Light Corn Syrup consists of corn syrup, salt, vanilla extract (vanilla beans, water, ethyl alcohol). The strawberry cream flavoring is dye-free and safe for the following allergies: milk, egg, soybeans, peanuts, tree nuts, wheat, grain, fish, shellfish, seeds, latex and some dyes. A 4 mL dose of placebo will be self-administered once. The study drug will be consumed within 5 minutes or less. There will be no observation period post dosing. No dose modifications will be necessary. Another dose will not be administered if the participants vomits after dosing.

<table>
<tr><th colspan="6">Regimen Description</th></tr>
<tr><th>Agent</th><th>Premedications; Precautions</th><th>Single Dose</th><th>Route</th><th>Schedule</th><th>Cycle Length</th></tr>
<tr><td>cannabidiol (Epidiolex) 100mg/mL</td><td>None</td><td>400 mg (4 mL; since Epidiolex’s concentration is 100mg/mL)</td><td>PO</td><td>Timepoint 1</td><td rowspan="2">Within 48 hours of CT or PET scan</td></tr>
<tr><td>Placebo</td><td>None</td><td>400 mg (4 mL)</td><td>PO</td><td>Timepoint 1</td></tr>
</table>

5.1.3 Instruments measuring study outcomes:

<u>Demographic questionnaire (Baseline):</u>
Variables of interest will be obtained via self-report and include: age, ethnicity, race, marital status, education, and occupational status.

<u>Cannabis History Questionnaire (Baseline):</u>
Cannabis use history will be obtained via self-report.

<u>Generalized Anxiety Disorder- 7 (GAD-7) (Baseline, Timepoint 1):</u> The widely used and validated GAD-7 measures anxiety severity on a 4-point Likert-type scale from not at all to

nearly every day. The presence of baseline anxiety, as measure by a GAD-7 score >4, is necessary for inclusion in this study.

<u>Patient Health Questionnaire (PHQ-9) (Baseline, Timepoint 1):</u> The widely used and validated PHQ-9 is a 9-item depression severity measure, with scores ranging from 0 to 27. Patients are asked to rate how they felt over the past two weeks using a 4-point Likert scale. Given that anxiety and depression are closely intertwined, this measure will help us to demonstrate that the active drug and placebo arms manifest similar levels of dysphoric distress. This measure will also help in screening to rule out "uncontrolled mental illness."

<u>VAMS (Timepoint 1, and Timepoint 2):</u> The Visual Analog Mood Scale (VAMS) a validated in-the-moment measure of eight specific mood states: Afraid, Confused, Sad, Angry, Energetic, Tired, Happy, and Tense. Each scale consists of a 100 mm vertical line connecting a neutral face and a face displaying a particular mood state (with an accompanying descriptor). Subjects are asked to mark their mood along this continuum. The measure is scored using a metric ruler and the "Afraid" subscale will serve as our primary endpoint. Other subscales will serve as exploratory endpoints.

<u>EORTC-QLQc30-nausea (Timepoint 1, and Timepoint 2):</u> The EORTC-QLQc30-nausea is a widely used and validated measure of symptom burden. It consists of 30-items, the first 28 of which employ a four-point scale (the final two are measured along a seven-point scale). Single item measures range in score from 0 to 100, with higher scores representing lower symptom burden. For the purposes of this study we will administer two items from the EORTC-QLQc30 on nausea and vomiting to assess a secondary endpoint.

<u>Numeric Pain Rating Scale (Baseline, Timepoint 1, Timepoint 2):</u> This widely used in-the-moment pain intensity scale is scored from 0 to 10 (with 10 representing "worst possible" pain). The NPRS will again help us to assess secondary endpoints.

<u>Medical Record Review (Baseline, Timepoint 3):</u> A member of the study team will record clinical data, including cancer diagnosis, disease stage, histology, and cancer treatments. Comorbidities or current medical history will also be extracted from medical records.

5.2. Pre-Treatment Criteria

5.2.1. Participants must have normal hepatic function at baseline as outlined below:

– total bilirubin	≤2 times institutional upper limit of normal (ULN)
– AST(SGOT)/ALT(SGPT)	≤3 × institutional ULN
– GAD-7 ≥5	

5.2.2. Cycle 1, Day 1

- Karnofsky ≥60%.

5.2.3. Subsequent Cycles

None

5.3.Agent Administration

Administration: Cannabidiol (Epidiolex), which comes in liquid form, is Federal Drug Administration-approved for rare forms of pediatric epilepsy. We will use an oral dose of 400 mg (4 mL) and will use this drug off-label for anxiety in adults with cancer. Dose administration is well within the recommended daily dose per package insert.

Dosing – This is a single dose study, with a dose of 400 mg (4 mL). Dose will be consumed within 5 minutes or less. Another dose will not be administered if the participants vomits after dosing. There will be no observation period post dosing.

Special Equipment – Disposable 10mL oral syringe with secured cap will be dispensed and either picked up, shipped, or delivered to the patient. The patient will then self-administer the cannabidiol (Epidiolex) using the oral syringe.

Oral Agents – Participants will be advised to avoid consuming high fat meals for one hour before and after study drug consumption. Examples of foods to avoid include those high in butter, cream, oil, cheese, whole milk, mayonnaise, salad dressing, jam, syrup, honey, nuts, as well as soft drinks, juices, sweets or desserts, pastas, breads, crackers, chips and meats. No other protocol specific procedures are necessary.

Caregiver Precautions – Study participants should not operate heavy machinery for 12 hours post study drug consumption.

5.3.1. CTEP and/or CIP IND Agent(s), or other IND agent

None

5.3.2. Other Agent(s)Placebo

Administration: The Dana-Farber Research Pharmacy will create the following non-sterile placebo liquid formulation: Karo Light Corn Syrup and FLAVORx strawberry cream flavoring in a ratio 20 ml:7 drops. We will use an oral dose of 4 mL.

Dosing – This is a single dose study, with a dose of 400 mg (4 mL). Dose will be consumed within 5 minutes or less. Another dose will not be administered if the participants vomits after dosing. There will be no observation period post dosing.

Special Equipment – A disposable 10mL oral syringe with secured cap will be dispensed and either picked up, shipped or delivered to the patient. The patient will then self-administer the placebo using the oral syringe.

Oral Agents – Participants will be advised to avoid consuming high fat meals for 1 hour before and after study drug consumption. Examples of foods to avoid include those high in butter, cream, oil, cheese, whole milk, mayonnaise, salad dressing, jam, syrup, honey, nuts, as well as

soft drinks, juices, sweets or desserts, pastas, breads, crackers, chips and meats. No other protocol specific procedures are necessary.

Caregiver Precautions – Study participants should not drive or operate machinery for 12 hours post study drug consumption.

5.3.3. *Other Modality(ies) or Procedures*

N/A

5.4. General Concomitant Medication and Supportive Care Guidelines

Because there is a potential for interaction of cannabidiol (Epidiolex) with other concomitantly administered drugs through the cytochrome P450 system, a licensed physician on the study team will review participants list of medication prior to study drug administration.

5.5. Criteria for Taking a Participant Off Protocol Therapy

The planned duration of therapy is a single day. Treatment will be halted in the setting of:

- Inability to ingest study medication on the day of study
- Intercurrent illness that prevents administration of treatment
- Unacceptable adverse event(s)
- Participant demonstrates an inability or unwillingness to comply with the oral medication regimen and/or documentation requirements

- Participant decides to withdraw from the protocol therapy
- General or specific changes in the participant's condition render the participant unacceptable for treatment in the judgment of the treating investigator

Participants will be removed from the protocol therapy when any of these criteria apply. The reason for removal from protocol therapy, and the date the participant was removed, must be documented in the case report form (CRF). Alternative care options will be discussed with the participant.

When a participant is removed from protocol therapy and/or is off of the study, the participant’s status must be updated in OnCore in accordance with REGIST-OP-1.

5.6. Duration of Follow Up

Participants will be followed for *approximately 1 week* after removal from protocol therapy or until death, whichever occurs first. Participants removed from protocol therapy for unacceptable adverse event(s) will be followed until resolution or stabilization of the adverse event.

5.7. Criteria for Taking a Participant Off Study

Participants will be removed from study when any of the following criteria apply:

- Lost to follow-up
- Withdrawal of consent for data submission
- Death
- Investigators discretion

The reason for taking a participant off study, and the date the participant was removed, must be documented in the case report form (CRF). In addition, the study team will ensure the participant's status is updated in OnCore in accordance with REGIST-OP-1.

There will also be a protocol around emergency unblinding. In this double-blind study, the research pharmacy not be blinded. Unblinding will occur when knowledge of the treatment is essential for the emergency management of a serious adverse event (SAE) as defined in section 7.1. Unblinding of the study for other reasons will be considered a major protocol violation. The PI must be notified if unblinding of a patient is necessary. The PI has designated the responsibility of unblinding to the MD's only. MD requesting this will notify and discuss with PI either by phone or email and when approved by the PI, the treating MD will notify the research pharmacy. An email will be sent to the research pharmacy at DFCIPharmacyIDS@partners.org with the protocol number, the patient name, MRN, the reason for the unblinding, and indicating the PI's approval. The DFCI research pharmacy will send the unblinded information back to the MD and receipt of the email will be acknowledged by the MD. During off hours, weekends, and holidays, the pharmacy administrator on call will be paged via the DFCI page operator at 617-632-3737 from within DFCI, or 617-632-3352 from outside DFCI.

6. DOSING DELAYS/DOSE MODIFICATIONS

This is a single dose study, therefore dosing delays are not allowed or applicable. There will be no modifications to the dose of cannabidiol (Epidiolex) or placebo given to study participants. Although there are recommended dose adjustments in individuals with hepatic dysfunction, these individuals are excluded from the study.

7. ADVERSE EVENTS: LIST AND REPORTING REQUIREMENTS

Adverse event (AE) monitoring and reporting is a routine part of every clinical trial. The following list of reported and/or potential AEs (Section 7.1) and the characteristics of an observed AE (Section 7.2) will determine whether the event requires expedited reporting **in addition** to routine reporting. Because this study is being completed in an advanced illness population, deaths due to cancer are expected. The research team will not report cancer-related deaths as adverse events.

The principal investigator will be responsible for monitoring and reporting all adverse events to the DFCI IRB for this study, but in the event that the principal investigator is unavailable, the co-investigators will assume this responsibility. The role of the responsible person is to 1) identify the concern, 2) develop the appropriate response to the concern with the use of consultants if necessary and 3) ensure proper reporting of concerns to the IRB.

All potential adverse events will be reported to the PI. The PI in consultation with Co-investigators will classify events according to level of severity. Details will be recorded on an adverse case report form as well as logged on a secure spreadsheet for reporting to the IRB.

The adverse event case report form will include a description of all undesirable experiences, required interventions, and an assessment of the subject after the event if possible. An estimate of the extent of injury, and prevention strategies will be reported. The principal investigator will classify the relationship of the study protocol to the event as follows:

- Not related: The event is clearly related to factors such as the subject's clinical state, not with the study protocol.
- Remote: The event was most likely related to factors such as the subject's clinical state, not with the study protocol.
- Possible: The event follows a reasonable temporal sequence associated with participating in the study and/or is consistent with events related to responding to queries about stress/drug craving but is possibly related to factors such as the subject's clinical state.
- Probable: The event follows a reasonable temporal sequence associated with participating in the study and/or is consistent with events related to dosing of cannabidiol (Epidiolex) in the study and cannot be reasonably explained by factors such as the subject's clinical state. The severity of an adverse event in both groups is defined as a qualitative assessment of the degree or intensity of an adverse event as determined by the principal investigator as follows:
  - Mild: No impact (in anyway) on the subjects.
  - Moderate: Impacts on the subject but is not life-threatening or incapacitating.
  - Severe: Fatal, life threatening, permanently disabling; severely incapacitating; requires/prolongs inpatient hospitalization.

7.1. Expected Toxicities

7.1.1. Adverse Events List(s) for cannabidiol (Epidiolex)

The most likely adverse event from single dose cannabidiol (Epidiolex) in this investigation is somnolence. Although we will ask study participants to refrain from benzodiazepine use between study visits 2 and 3, participants may still use benzodiazepines while not under the study team supervision. Combination of benzodiazepine plus cannabidiol (Epidiolex) may result in somnolence. No reports of hypopnea or respiratory arrest have been described with cannabidiol (Epidiolex). Please refer to attached commercial package insert for details and comprehensive list of adverse events. They include:

- nonfatal hepatotoxicity
- seizures
- suicidal ideation or behavior
- nausea and vomiting

7.1.2. Adverse Events List(s) for Placebo

- none

7.1.3. Adverse Events List(s) for PHQ-9

- Any participant who evidences potential suicidal ideation on the PHQ-9 will be evaluated by one of the study's psychiatrists or referred a psycho-oncologist in the Department of Psychosocial Oncology and Palliative Care for such clinical evaluation.

7.2. Adverse Event Characteristics

- **CTCAE term (AE description) and grade:** The descriptions and grading scales found in the revised NCI Common Terminology Criteria for Adverse Events (CTCAE) version 5.0 will be utilized for AE reporting. All appropriate treatment areas should have access to a copy of the CTCAE version 5.0. A copy of the CTCAE version 5.0 can be downloaded from the CTEP web site http://ctep.cancer.gov/protocolDevelopment/electronic_applications/ctc.htm.

- **For expedited reporting purposes only:**
  - AEs for the agent(s) that are listed above should be reported only if the adverse event varies in nature, intensity or frequency from the expected toxicity information which is provided.
  - Other AEs for the protocol that do not require expedited reporting are outlined in the next section (Expedited Adverse Event Reporting) under the sub-heading of Protocol-Specific Expedited Adverse Event Reporting Exclusions.

- **Attribution** of the AE:
  - Definite – The AE *is clearly related* to the study treatment.
  - Probable – The AE *is likely related* to the study treatment.
  - Possible – The AE *may be related* to the study treatment.
  - Unlikely – The AE *is doubtfully related* to the study treatment.
  - Unrelated – The AE *is clearly NOT related* to the study treatment.

7.3. Adverse Event Reporting

7.3.1. In the event of an unanticipated problem or life-threatening complications treating investigators must immediately notify the Overall PI.

7.3.2. Investigators **must** report to the Overall PI any adverse event (AE) that occurs after the single dose of study drug, or within 30 days of the single dose of study drug on the local institutional SAE form.

7.3.3. DF/HCC Adverse Event Reporting Guidelines

Investigative sites within DF/HCC will report AEs directly to the DFCI Office for Human Research Studies (OHRS) per the DFCI IRB reporting policy.

7.3.4. Protocol-Specific Adverse Event Reporting Exclusions

For this protocol only, the AEs/grades listed below do not require expedited reporting to the Overall PI or the DFCI IRB. However, they still must be reported through the routine reporting mechanism (i.e., case report form).

CTCAE SOC	Adverse Event	Grade	Hospitalization/ Prolongation of Hospitalization	Attribution	Comments
**Allergic Reaction**		**1,2**			
**Alanine aminotransferase increased**		**1, 2**			
**Aspartate aminotransferase increased**		**1, 2**			
**Ataxia**		**1, 2**			
**Cognitive disturbance**		**1**			
**Dizziness**		**1, 2**			
**Lethargy**		**1**			
**Agitation**		**1, 2**			
**Confusion**		**1**			
**Depression**		**1, 2**			

7.4. Reporting to the Food and Drug Administration (FDA)

Not applicable since FDA deemed this IND exempt.

7.5. Reporting to Hospital Risk Management

Participating investigators will report to their local Risk Management office any participant safety reports, sentinel events or unanticipated problems that require reporting per institutional policy.

7.6. Routine Adverse Event Reporting

All Adverse Events **must** be reported in routine study data submissions to the Overall PI on the toxicity case report forms. **AEs reported through expedited processes (e.g., reported to the IRB, FDA, etc.) must also be reported in routine study data submissions.**

8. PHARMACEUTICAL INFORMATION

A list of the adverse events and potential risks associated with the investigational or other agents administered in this study can be found in Section 7.1.

8.1. Cannabidiol (Epidiolex)

8.1.1. Description

Cannabidiol (Epidiolex) is an herbal-derived phytocannabinoid. The molecular formula is $C_{21}H_{30}O_2$. The molecular weight is 314.46. After oral administration, the time to maximum concentration (Cmax) is 2.5-5 hours. In individuals who have recently ingested a high fat diet, the Cmax increases by 5-fold. Cannabidiol is protein-bound but has a volume of distribution of 20963-32849L. After twice daily dosing, the half-life of cannabidiol is 56-61 hours. Cannabidiol is hepatically metabolized via CYP2C19 and 3A4 to 7-OH-CBD. 7-OH-CBD is the major metabolite and remains pharmacologically active. Metabolites of cannabidiol undergo fecal-elimination. A secondary route of elimination is renal. Cannabidiol is a substrate for CYP 2C19 and 3A4 and inhibitor of 1A2 and 2B6. A minor metabolite, 7-COOH-CBD is a PgP substrate. Major drug interactions include concomitant use of clobazam and valproic acid. Coadministration of clobazam with CBD results in increased 7-OH-CBD levels. Coadministration of valproic acid with CBD has no effect on valproate levels, but the combination may result in reversible hepatic injury.

8.1.2. Form

Cannabidiol (Epidiolex), manufactured by GW Pharmaceuticals, is a clear (colorless to yellow) solution intended for oral consumption. In addition to CBD (100 mg/mL), it contains sesame seed oil, sucralose, alcohol and strawberry flavor.

8.1.3. Storage and Stability

Cannabidiol (Epidiolex) should be stored at room temperature 20 to 25°C (68 to 77 °F) in its original bottle and in an upright position. Excursions are permitted between 15 to 30°C (59 to 86°F). Once opened, Cannabidiol has a shelf life of 84 days. The oral solution may be kept in the syringe for up to 24 hours. For this reason the medication will be overnighted to patients.

8.1.4. Compatibility

Not Applicable

8.1.5. Handling

This is commercially available and no special handling considerations are necessary.

8.1.6. Availability

Cannabidiol (Epidiolex) is commercially available and the DFCI pharmacy will purchase the drug on behalf of our research team. The study will cover the cost for Epidiolex.

8.1.7. Preparation

Cannabidiol (Epidiolex) at the appropriate dose will loaded into syringe labeled "Cannabidiol or Placebo" (or something similar).

8.1.8. Administration

Disposable 10mL oral syringe will be used to administer cannabidiol (Epidiolex) PO at the conclusion of Timepoint 1.

8.1.9. Ordering

Cannabidiol (Epidiolex), which is commercially available, will be obtained through GW Pharmaceuticals. The study drug will be shipped to and logged at the investigational pharmacy at DFCI. The research pharmacy will dispense the study drug either directly to the participant or to the study staff for mailing. Patient drug accountability will be ensured through self-report in the form of query on Study Day Timepoint 2 (Appendix H).

8.1.10. Accountability

The investigator, or a responsible party designated by the investigator, will maintain a careful record of the inventory and disposition of the agent using the NCI Drug Accountability Record Form (DARF) or another comparable drug accountability form. (See the NCI Investigator's Handbook for Procedures for Drug Accountability and Storage.)

8.1.11. Destruction and Return

Expired or unused supplies of cannabidiol or placebo will be destroyed according to institutional guidelines. Destruction will be documented in the Drug Accountability Record Form. If the study drug is in possession of the study participant and they are unable to take the drug during the time period. We will ask the participant to return the unused investigational drug for destruction and log this on our internal drug tracking log.

8.2. Placebo

8.2.1. Description

The Dana-Farber Research Pharmacy will create the placebo as follows: a non-sterile formula of Karo Light Corn Syrup and FLAVORx strawberry cream flavoring in a ratio 20 ml:7 drops. Karo Light Syrup consists of salt, vanilla extract (vanilla beans, water, ethyl alcohol).

8.2.2. Form

Formulated by the Dana-Farber Research Pharmacy, the placebo is a non-sterile, caramel-colored solution intended for oral consumption, containing Karo Light Corn Syrup and FLAVORx strawberry cream flavoring in a ratio 20 mL:7 drops. The strawberry cream flavoring is dye-free and safe for the following allergies: milk, egg, soybeans, peanuts, tree nuts, wheat, grain, fish, shellfish, seeds, latex and some dyes.

8.2.3. Storage and Stability

Placebo should be stored at room temperature (68 to 77 F) in a bottle kept in an upright position. FLAVORx has an expiry date on the stock bottle. The oral solution may be kept in the syringe for up to 24 hours. For this reason the medication will be overnighted to patients.

8.2.4. Compatibility

Not applicable

8.2.5. Handling

No special handling considerations are necessary.

8.2.6. Availability

The Dana-Farber Research Pharmacy will purchase the components of the placebo, which are commercially available, on behalf of our research team. The study will cover the cost for placebo.

8.2.7. Preparation

An appropriate dose of the placebo will be loaded into syringe labeled “Cannabidiol or Placebo” (or something similar).

8.2.8. Administration

Disposable 10mL oral syringe will be used for patients to self-administer placebo PO.

8.2.9. Ordering

Components of the placebo are commercially available, and various manufacturers may be used to purchase the product by Dana-Farber Research Pharmacy. The components of the placebo will be logged at the investigational pharmacy at DFCI. The research pharmacy will dispense the placebo either directly to the participant or to the study staff for mailing. Patient drug accountability will be ensured through self-report in the form of query on Study Day Timepoint 2 (Appendix H).

8.2.10. Accountability

The investigator, or a responsible party designated by the investigator, will maintain a careful record of the inventory and disposition of the agent using the NCI Drug Accountability Record Form (DARF) or another comparable drug accountability form. (See the NCI Investigator's Handbook for Procedures for Drug Accountability and Storage.)

8.2.11. Destruction and Return

Expired or unused supplies of cannabidiol or placebo will be destroyed according to institutional guidelines. Destruction will be documented in the Drug Accountability Record Form.

9. STUDY CALENDAR

	Baseline assessment	Timepoint 1 Treatment day, pre-assessment	Timepoint 2 Treatment day, post-assessment	Timepoint 3 Follow-up assessment
	Prior to Timepoint 1	Within 48 hours of a CT/PET scan to assess tumor burden	3 (+/- 1) hour post ingestion	≤1-week (+/- 7 days) post ingestion
**Background Information**				
Informed consent***	X			
Screener questionnaire	X			
Demographics	X			
Medical history	X			X
Concurrent medications	X	X		
Performance status	X			
LFTs	X			
bHCG**	X			
GAD-7	X	X		
PHQ9	X	X		
Cannabis History Questionnaire	X			
Cannabidiol (Epidiolex) vs. Placebo		X*		
**Confirmation of study drug consumption**			X	
**Patient Provisions**				
$50 gift card				X
**Primary endpoints**				
VAMS afraid subscale		X	X	
**Secondary endpoints**				
NPRS	X	X	X	
EORTC-QLCc30-nausea		X	X	
Other subscales of VAMS		X	X	
Qualitative interview				X
**Safety Monitoring**				
Adverse Event Evaluation		X	X	

*** = Study drug to be administered after completion of Treatment day, pre-assessments**
****= bHCGs will be collected on all potential participants under the age of 54-years, unless known to be post-menopausal or status post hysterectomy**
***** = to be completed with physician involvement**

10. DATA REPORTING / REGULATORY REQUIREMENTS

Adverse event lists, guidelines, and instructions for AE reporting can be found in Section 7.0 (Adverse Events: List and Reporting Requirements).

10.1. Data Reporting

10.1.1. Method

The study staff will collect, manage, and perform quality checks on the data for this study. This study will be monitored by the Clinical Data Update System (CDUS) Version 3.0. Cumulative protocol- and participant-specific CDUS data will be submitted electronically to CTEP on a quarterly basis, either by FTP burst of data or via the CDS web application. Reports are due January 31, April 30, July 31, and October 31. Instructions for submitting data using the CDUS can be found on the CTEP Web site (http://ctep.cancer.gov/reporting/cdus.html).

10.1.2. Responsibility for Data Submission

Participant institutions are responsible for submitting CDUS data and/or data forms to either the Coordinating Center or to the Lead Organization on the study quarterly. The date for submission to the Coordinating Center or to the Lead Organization will be set by them. CDUS does not accept data submissions from the participant institutions on the study. When setting the dates, allow time for Coordinating Center compilation, Overall PI review, and timely submission to CTEP by the quarterly deadlines (see Section 12.1.1). For trials monitored by CTMS, a quarterly report of data will be provided by Theradex to the Coordinating Center.

Either the Coordinating Center or the Lead Organization is responsible for compiling and submitting CDUS data to CTEP for all participant institutions and for providing the data to the Overall PI for review.

10.2. Data Safety Monitoring

The DF/HCC Data and Safety Monitoring Board (DSMB) will review and monitor study progress, toxicity, safety and other data from this study. The Board is chaired by a medical oncologist from outside of DF/HCC and its membership composed of internal and external institutional representation. Information that raises any questions about participant safety or protocol performance will be addressed by the Overall PI, statistician and study team. Should any major concerns arise, the DSMB will offer recommendations regarding whether or not to suspend the study.

The DSMB will meet twice a year to review accrual, toxicity, response and reporting information. Information to be provided to the DSMB may include: participant accrual; treatment regimen information; adverse events and serious adverse events reported by category; summary of any deaths on study; audit results; and a summary provided by the study team. Other information (e.g. scans, laboratory values) will be provided upon request.

11. STATISTICAL CONSIDERATIONS

This is a phase II, randomized, placebo-controlled study to assess the preliminary efficacy of offering a single dose of CBD versus placebo in reduction of anxiety in patients with advanced (stage IV) solid tumor anticipating CT to assess tumor burden. Eligible patients must have measurable anxiety-- at least “mild” anxiety as measured by the GAD-- at the pre-screen visit.

Anxiety level at each of the time points (pre-screening visit, pre-dose, and 3[+/-1] hour post dose) will be plotted for each study group. The paired change (pre-dose to post-dose) in anxiety will be estimated along with a 95% confidence interval for each study group. The change in anxiety will be compared by study group with a Wilcoxon rank sum test. The side-effect profile, as measured by the PRO-CTCAE will be tabled according to side effect. Patient reported experience with CBD and the acceptability will be summarized with descriptive statistics and qualitative methodology as appropriate

11.1. Study Design/Endpoints

Primary Endpoint

Change in anxiety will be assessed by the Visual Analog Mood Scale (VAMs) anxiety subscale, ascertained on the day of the drug administration pre-dose (T1) and 3(+/- 1) hour after drug administration (T2). The change in anxiety score will be calculated for each patient and a Wilcoxon rank sum test will be used to assess whether the change score is different among patients randomized to CBD as compared to placebo.

Secondary and Exploratory Endpoints

Secondary and exploratory objectives include a description of the side-effect profile of CBD in oncology patients, patient reported experience and acceptability of CBD, and the estimation of the change in state anxiety as well as other VAMs subscales (Afraid, Confused, Sad, Angry, Energetic, Tired, Happy, and Tense). Secondary endpoint analyses will be considered exploratory (no alpha adjustment given the relatively small sample size) and will provide context for interpretation of the primary endpoint.

Randomization

Patients will be randomized 2:1 to CBD or placebo using permuted blocks within strata (one single stratification variable, baseline anxiety with 2 levels, will be used) and a double-blind procedure will be followed. Randomization will be completed by the Office of Data Quality (ODQ). ODQ will send the randomization assignment to the DFCI Research Pharmacy and the pharmacy will dispense the medication. The justification for the 2:1 randomization is to better assess side-effects and to allow for more qualitative data at the completion of the study.

11.2. Sample Size, Accrual Rate and Study Duration

Accrual Targets					
**Ethnic Category**	**Sex/Gender**				
	**Females**		**Males**		**Total**
Hispanic or Latino	8	+	0	=	8
Not Hispanic or Latino	192	+	0	=	192
**Ethnic Category: Total of all subjects**	200 (A1)	+	0 (B1)	=	200(C1)
**Racial Category**					
American Indian or Alaskan Native	0	+	0	=	0
Asian	4	+	0	=	4
Black or African American	4	+	0	=	4
Native Hawaiian or other Pacific Islander	0	+	0	=	0
White	192	+	0	=	192
**Racial Category: Total of all subjects**	200 (A2)	+	0 (B2)	=	200(C2)
	(A1 = A2)		(B1 = B2)		(C1 = C2)

The Visual Analog Mood Scale (VAMs) anxiety subscale is measured on a 0 to 100 scale and is then converted to a T-score standardized by age and gender. The VAMs manual suggests that, in a pre-test/post-test setting, a score differing by 20T indicates a reliable change in mood and a score differing by 30T indicates a reliable and clinically meaningful change in mood[20]. Studies comparing depressed and non-depressed psychiatric patients indicated possible standard deviations ranging from 15-36[20]. Additionally, a previous study[18] demonstrated that the change from pre-dose to 140 minutes post-dose in the VAMs anxiety subscale was approximately 16.7 for CBD and 3.5 for placebo respectively in 10 subjects with social anxiety disorder. In the same sample, the reported standard deviations pre- and post- CBD ranged from approximately 7 to 15.

A two-sided, Wilcoxon rank sum test will be used to assess the difference in change in anxiety score between CBD and placebo. Estimating a minimal change in anxiety for patients assigned to placebo, a difference in the change score of 20 is be targeted. Assuming a two-sided significance level of 0.05, a 2 to 1 randomization, and a standard deviation of 20, there is 80% power to detect a difference of 20 (effect size = 1) in the change in anxiety score between CBD and placebo with 45 patients (30 CBD, 15 placebo). Based on an approximated 10% attrition rate, 50 patients will be enrolled and randomized.

11.3. Stratification Factors

Randomization will be stratified on baseline anxiety level, mild to moderate (5-14) versus severe (15-21).

11.4. Interim Monitoring Plan

N/A

11.5. Analysis of Primary Endpoints

Please see above.

11.6. Analysis of Secondary Endpoints

This will be an intent-to-treat analysis. Secondary endpoint analyses will be considered exploratory and will provide context for interpretation of the primary endpoint.

11.7. Reporting and Exclusions

11.7.1. Evaluation of Toxicity

All participants will be evaluable for toxicity from the time of their first treatment.

11.7.2. Evaluation of the Primary Efficacy Endpoint

Sub-analyses may then be performed on the basis of a subset of participants, excluding those for whom major protocol deviations have been identified (e.g., early death due to other reasons, early discontinuation of treatment, major protocol violations, etc.). However, these sub-analyses will not serve as the basis for drawing conclusions concerning treatment efficacy, and the reasons for excluding participants from the analysis will be clearly reported. If applicable to the endpoint, the 95% confidence intervals will also be provided.

12. PUBLICATION PLAN

The results will be made public within 24 months of reaching the end of the study. The end of the study is the time point at which the last data items are to be reported, or after the outcome data are sufficiently mature for analysis, as defined in the section on Sample Size, Accrual Rate and Study Duration. If a report is planned to be published in a peer-reviewed journal, then that initial release may be an abstract that meets the requirements of the International Committee of Medical Journal Editors. A full report of the outcomes should be made public no later than three (3) years after the end of the study.

At the end of the trial, PI will contact ODQ/research pharmacy and obtain randomization assignments.

REFERENCES

1. Administration FaD. *Highlights of Prescribing Information.* 2018 2018.
2. Yeshurun M, Shpilberg O, Herscovici C, et al. Cannabidiol for the prevention of graft-versus-host-disease after allogeneic hematopoietic cell transplantation: results of a phase II study. 2015;21(10):1770-1775.
3. Blessing EM, Steenkamp MM, Manzanares J, Marmar CRJN. Cannabidiol as a Potential Treatment for Anxiety Disorders. *Neurotherapeutics.* 2015;12(4):825-836.
4. Borgelt LM, Franson KL, Nussbaum AM, Wang GS. The Pharmacologic and Clinical Effects of Medical Cannabis. *Pharmacotherapy.* 2013;33(2):195-209.
5. de Mello Schier AR, de Oliveira Ribeiro NP, e Silva ACdO, et al. Cannabidiol, a Cannabis Sativa Constituent, as an Anxiolytic Drug. *Official Journal of the Brazilian Psychiatric Association.* 2012;34:1.
6. Mechoulam R, Parker LA, Gallily R. Cannabidiol: An Overview of Some Pharmacological Aspects. *Journal of Clinical Pharmacology.* 2002;42(S1):11S-19S.
7. Bergamaschi MM, Queiroz RHC, Chagas MHN, et al. Cannabidiol Reduces the Anxiety Induced by Simulated Public Speaking in Treatment-Naïve Social Phobia Patients. *Neuropsychopharmacology.* 2011;36:1219.
8. Fusar-Poli P, Crippa JA, Bhattacharyya S, et al. Distinct Effects of Δ9-Tetrahydrocannabinol and Cannabidiol on Neural Activation During Emotional Processing. *Archives of General Psychiatry.* 2009;66(1):95-105.
9. Linden W, Vodermaier A, MacKenzie R, Greig D. Anxiety and Depression After Cancer Diagnosis: Prevalence Rates by Cancer Type, Gender, and Age. *Journal of Affective Disorders.* 2012;141(2):343-351.
10. Bauml JM, Troxel, A., Epperson, C.N., Cohen, R.B., Schmitz, K., Stricker, C., Shulman, L.N., Bradbury, A., Mao, J.J. and Langer, C.J. Scan-associated distress in lung cancer: Quantifying the impact of "scanxiety". *Lung Cancer.* 2016;100:110-113.
11. Thompson CA, Charlson M, Schenkein E, et al. Surveillance CT scans are a source of anxiety and fear of recurrence in long-term lymphoma survivors. *Annals of Oncology.* 2010;21(11):2262-2266.
12. Derogatis LR, Feldstein M, Melisaratos N, et al. A Survey of Psychotropic Drug Prescriptions in an Oncology Population. *Cancer.* 1979;44(5):1919-1929.
13. Agarwal SD, Landon BE. Patterns in Outpatient Benzodiazepine Prescribing in the United StatesPatterns in Outpatient Benzodiazepine Prescribing in the United StatesPatterns in Outpatient Benzodiazepine Prescribing in the United States. *JAMA Network Open.* 2019;2(1):e187399-e187399.
14. Shead DA, Corrigan A, Kidney S, Hanisch LJ, Clarke R. *Nausea and Vomiting.* NCCN;2016.
15. Institute D-FC. Preparing for your PET/CT Scan. *Health Library* 2018; https://www.dana-farber.org/health-library/articles/preparing-for-your-petct-scan/. Accessed 02/13/2019, 2019.
16. Grlie LJBoN. A Comparative Study on Some Chemical and Biological Characteristics of Various Samples of Cannabis Resin. *Bulletin on Narcotics.* 1976;14:37-46.
17. Cunha JM, Carlini EA, Pereira AE, et al. Chronic Administration of Cannabidiol to Healthy Volunteers and Epileptic Patients. *Pharmacology.* 1980;21(3):175-185.
18. Zuardi AW, Morais SL, Guimarães FS, Mechoulam R. Antipsychotic Effect of

Cannabidiol. *The Journal of Clinical Psychiatry*. 1995;56(10):485-486.

19. Abreu C, Grilo, A., Lucena, F. and Carolino, E. Oncological patient anxiety in imaging studies: the PET/CT example. *Journal of Cancer Education.* 2017;32(4):820-826.
20. Stern RA, Arruda JE, Hooper CR, Wolfner GD, Morey CE. Visual Analogue Mood Scales to Measure Internal Mood State in Neurologically Impaired Patients: Description and Initial Validity Evidence. *Aphasiology*. 1997;11(1):59-71.
