# Supplementary material for: Cannabidiol for Scan-Related Anxiety in Women With Advanced Breast Cancer: A Randomized Clinical Trial
Source: JAMA Netw Open. 2024 Dec 16;7(12):e2450391. doi: 10.1001/jamanetworkopen.2024.50391 (PMC11650394; doi:10.1001/jamanetworkopen.2024.50391)
Supplement: Supplement 2. — Data Sharing Statement [file jamanetwopen-e2450391-s002.pdf]

# Data Sharing Statement

Nayak. Cannabidiol for Scan-Related Anxiety in Women With Advanced Breast Cancer. *JAMA Netw Open*. Published December 16, 2024. doi:10.1001/jamanetworkopen.2024.50391

**Data**
**Additional Information:** ClinicalTrials.gov https://clinicaltrials.gov/study/NCT04482244?id=NCT04482244&rank=1 ID NCT04482244
**Data available:** No

**Additional Information**
**Explanation for why data not available:** Any reasonable request for data will be granted.
